# Simultaneous analysis of all single-nucleotide polymorphisms in genome-wide association study of rheumatoid arthritis

**DOI:** 10.1186/1753-6561-3-s7-s11

**Published:** 2009-12-15

**Authors:** George Mathew, Hongyan Xu, Varghese George

**Affiliations:** 1 Department of Mathematics, Missouri State University, 901 South National Avenue, Springfield, MO 65897, USA; 2 Department of Biostatistics, Medical College of Georgia, 1120 15th Street, Augusta, GA 30912, USA

## Abstract

The availability of very large number of markers by modern technology makes genome-wide association studies very popular. The usual approach is to test single-nucleotide polymorphisms (SNPs) one at a time for association with disease status. However, it may not be possible to detect marginally significant effects by single-SNP analysis. Simultaneous analysis of SNPs enables detection of even those SNPs with small effect by evaluating the collective impact of several neighboring SNPs. Also, false-positive signals may be weakened by the presence of other neighboring SNPs included in the analysis. We analyzed the North American Rheumatoid Arthritis Consortium data of Genetic Analysis Workshop 16 using HLasso, a new method for simultaneous analysis of SNPs. The simultaneous analysis approach has excellent control of type I error, and many of the previously reported results of single-SNP analyses were confirmed by this approach.

## Background

The increase in genome-wide experiments and sequencing of multiple genomes has resulted in the availability of large data sets for genome-wide association studies (GWAS) of complex diseases. The usual procedure is to test these single-nucleotide polymorphisms (SNPs) one by one for association with the disease status. This means one will be testing the marginal effect of a SNP on a disease without consideration of any other interacting SNPs in the model. This approach will inherently increase the overall probability of false positives [1]. Because complex diseases arise from many, possibly interacting, genes from the genome, it would be more appropriate to study the effect of several genes jointly rather than testing each of them separately [2]. The single-SNP analysis may ignore information provided by a joint distribution [3].

The North American Rheumatoid Arthritis Consortium (NARAC) data of Genetic Analysis Workshop (GAW) 16 provides an excellent opportunity to analyze simultaneously a very large collection of SNPs for GWAS. We adopt a recently developed method [4] for the simultaneous analysis of SNPs from the NARAC data. This method is useful when the number of SNPs is much greater than the number of individuals in a case-control study. The procedure is formulated as a problem of variable selection in a logistic regression framework by treating each SNP as a covariate. It attempts to find a collection of SNPs to obtain the "best" model to explain the disease status for a specified error rate.

## Methods

The NARAC data consists of 868 cases of rheumatoid arthritis (RA) and 1194 controls. The software program HLasso [4] was employed for the simultaneous analysis of SNPs. The program adopts a Bayesian approach for logistic regression and makes use of the normal-exponential-gamma (NEG) probability density function as the penalty function (see Hoggart et al. [4] for details). The NEG probability density function has two parameters, a scale parameter *γ *and a shape parameter *η*. As both *γ *and *η *increase such that /*γ *remains a constant, say *λ*, the NEG converges to the double exponential distribution with rate parameter *λ*. The predictors in the logistic regression model are the SNP genotypes, coded as 0, 1, and 2, corresponding to an additive model. These coded values are standardized to have mean zero and unit variance. The procedure searches for a collection of SNPs for which the posterior mode is positive. A positive posterior mode indicates a signal of association that is strong enough to overcome the prior preference of zero effect, and the corresponding set of SNPs are declared as significantly associated with the disease.

Further analysis was done to identify whether the SNPs declared significant by this method confirms the earlier findings by single-marker analyses. We examined whether the markers identified as significant by the simultaneous approach are in high linkage disequilibrium (LD) with SNPs that are already identified to be associated with the disease. Particular attempt was made to test SNPs identified by single-marker analysis on chromosomes 1, 6, and 9 from other data sets. The software package Haploview [5] was used to compute the pair-wise LD for SNPs on the chromosomes.

## Results

Chromosomes 1 through 22 were analyzed. Table 1 provides the number of SNPs found to be significant on each chromosome. From a total of 531,689 SNPs, 2627 SNPs were found to be significantly associated with the disease by this procedure, yielding an upper bound of 0.4941% for the empirical type I error rate.

**Table 1:** Number of significant markers identified by the simultaneous analysis and markers with extreme effect sizes

Chromosome number	Number of SNPs	Number of significant SNPs	Markers with largest risk and protective effects			
	
			Largest risk effect		Largest protective effect	
		
			Marker	Effect size	Marker	Effect size
1	40929	142	rs7519615	1.73638	rs2275864	-1.41482
2	44090	140	rs655783	0.91779	rs938869	-2.3145
3	36690	138	rs7616866	202.447	rs9288967	-4.23478
4	32628	133	rs768063	155.04	rs6816684	-2.31044
5	33612	90	rs344156	1.46745	rs6899062	-1.68164
6	35574	82	rs6935937	3.06767	rs1856363	-2.18496
7	29244	130	rs9632680	96.105	rs4732523	-2.41344
8	30990	130	rs7006628	0.577914	rs2853259	-1.50527
9	26128	125	rs6478815	154.845	rs1387292	-3.12822
10	28331	131	rs2388121	0.777184	rs1909668	-1.36459
11	26477	119	rs1879445	123.081	rs7932437	-6.04313
12	26365	129	rs1146114	1.43868	rs7300982	-1.59685
13	20242	129	rs2323883	4.76091	rs9740397	-3.53682
14	17951	124	rs4296166	1.53678	rs4983565	-2.02512
15	16166	121	rs7183817	0.814795	rs3743372	-2.60765
16	16460	111	rs4238802	0.66247	rs4783187	-1.98159
17	14027	123	rs2880328	92.8919	rs9747823	-1.31507
18	16450	123	rs2303508	78.2526	rs1982040	-2.32656
19	9236	101	rs3810256	1.11899	rs10404348	-3.04764
20	13843	114	rs6513195	3.13637	rs6138601	-4.63256
21	8051	102	rs2823819	0.971309	rs1539902	-1.08918
22	8205	90	rs761917	1.46745	rs17406434	-1.68164

Total	531689	2627				

The logistic regression model, logit(P(affected)) = , was employed for the analyses. When a marker is declared significant, the estimate  of *β *provides an estimate of the effect of that marker on the affection status of the disease. An estimate of the odds ratio (OR) for the disease is exp(). This means that OR > 1 if  > 0, and OR < 1 if the  < 0. Therefore, when  > 0, the allele coded as "1" increases the disease risk; and when  < 0, the allele coded as "1" has a protective effect and decreases the disease risk. Table 1 also provides the marker with the largest risk effect value and the marker with the largest protective effect value on each chromosome.

It is well known that HLA-DRB1 region on chromosome 6 is associated with RA. In the HLA-DRB1 region the marker rs602875 at position 32.68 Mb was identified to be significant in our study. By single-SNP analysis of a different data set, Chang et al. identified the marker rs1953126 near the TRAF1-C5 region on chromosome 9 to be associated with RA [6]. Also, Plenge et al., through single SNP analysis, identified the markers rs3761847 and rs2900180 in the same region to be of risk for RA [7]. In our simultaneous analysis, the marker rs3933326 near the TRAF1-C5 region was identified to be significant, which is in high LD with the markers rs1953126, rs3761847, and rs2900180. Begovich et al. [8] identified the marker rs2476601 in the PTPN22 region of chromosome 1 to be associated with risk for RA. The simultaneous analysis also identified this marker to be associated with RA, confirming the earlier findings.

## Discussion

The type I error is given by , where *f' *is the derivative of -log(NEG) at the origin (which is the derivative of the double exponential distribution with parameter *γ *= /*γ *at the origin), *n*_0 _is the number of cases, *n*_1 _is the number of controls, and Φ is the cumulative distribution function of the standard normal distribution [4]. For our computations, we set *λ *to be 50 and the shape parameter *η *to be 0.1, so that the calculated value of *α *= 0.025. The simultaneous analysis identified only 0.4941% of SNPs to be significantly associated with RA. Because the vast majority of the SNPs are not associated with RA, this is a conservative estimate of the empirical type I error. Comparing this with the nominal level of 0.025, it is evident that the simultaneous analysis procedure has excellent control of type I error.

In modeling all SNPs simultaneously, the HLasso program includes a SNP in the model if it significantly improves prediction of case-control status beyond that obtained from SNPs already included in the model [4]. Problems could arise when there are two or more tightly linked causal markers. In this case, if HLasso chooses only one marker, its effect could be inflated. For example, the marker rs7616866 on chromosome 3 at position 64317576 had the largest effect size 202.447, and was found to be significant. The LD values, *D'*, of this marker with the markers rs9812599 at 64315677, rs7615058 at 64321040, rs6445398 at 64309797, and rs1860819 at 64323994 are 0.857, 0.769, 1.0, and 0.977, respectively. When there is high pair-wise LD between markers, multicollinearity is present, and in such cases it is not possible to make meaningful statements about individual effect sizes. However, the overall model is valid in predicting the disease status of the disease.

The simultaneous analysis of SNPs confirmed several previous findings of causal variants for RA: the HLA-DRB1 region of chromosome 6, the TRF1-C5 region on chromosome 9, and the PTPN22 region on chromosome 1. However, it did not confirm the findings on the PADI4 region on chromosome 1, known to be a causal variant for RA [10].

We did not check for population stratification because the samples were from Caucasian population, which is expected to be relatively homogeneous. However, Sarasu et al. [9] suggests that population stratification is present in the data. The method we use seems to be reasonably robust to population stratification because type I error is well controlled. The SNP data had already been checked for errors [11], and hence, no further checking for errors was done. There were many significant markers on each chromosome. We checked whether these markers were themselves in high LD with each other. Five pairs among them on chromosome 6 were found to be in high LD, and none were found on any other chromosomes. It could be due to the fact that HLasso program includes a SNP in the model if it significantly improves prediction of case-control status beyond that obtained from the SNP already included. If there are two strongly correlated predictors, and if the marginal increase is minimal, then the program is inclined to choose only one of the two predictors.

## Conclusion

The simultaneous analysis of all SNPs using HLasso reduced considerably the overall probability for false positives. The method confirmed many of the previous findings by the single-SNP analysis.

## List of abbreviations used

GWAS: Genome-wide association studies; LD: Linkage disequilibrium; NARAC: North American Rheumatoid Arthritis Consortium; NEG: Normal-exponential-gamma; OR: odds ratio; RA: Rheumatoid arthritis; SNP: Single-nucleotide polymorphism

## Competing interests

The authors declare that they have no competing interests.

## Authors' contributions

GM performed the analysis and drafted the manuscript. HX conceived of the study, participated in the analysis and helped to draft the manuscript. VG participated in the design and coordination of the study. All authors read and approved the final manuscript.
